# *Trichomonas stableri* n. sp., an agent of trichomonosis in Pacific Coast band-tailed pigeons (*Patagioenas fasciata monilis*)^☆^

**DOI:** 10.1016/j.ijppaw.2013.12.002

**Published:** 2013-12-28

**Authors:** Yvette A. Girard, Krysta H. Rogers, Richard Gerhold, Kirkwood M. Land, Scott C. Lenaghan, Leslie W. Woods, Nathan Haberkern, Melissa Hopper, Jeff D. Cann, Christine K. Johnson

**Affiliations:** a Wildlife Health Center, School of Veterinary Medicine, University of California, Davis, CA 95616, United States; b Wildlife Investigations Laboratory, California Department of Fish and Wildlife, Rancho Cordova, CA 95670, United States; c Department of Biomedical and Diagnostic Sciences, University of Tennessee, Knoxville, TN 37996, United States; d Department of Biological Sciences, University of the Pacific, Stockton, CA 95211, United States; e Center for Renewable Carbon, University of Tennessee, Knoxville, TN 37996, United States; f California Animal Health and Food Safety Laboratory, University of California, Davis, CA 95616, United States; g California Department of Fish and Wildlife, Monterey, CA 93940, United States

**Keywords:** Avian trichomonosis, *Trichomonas gallinae*, Columbidae, Band-tailed pigeon, Phylogeny, ITS1/5.8S/ITS2, Fe-hydrogenase, *rpb1*

## Abstract

*Trichomonas gallinae* is a ubiquitous flagellated protozoan parasite, and the most common etiologic agent of epidemic trichomonosis in columbid and passerine species. In this study, free-ranging Pacific Coast band-tailed pigeons (*Patagioenas fasciata monilis*) in California (USA) were found to be infected with trichomonad protozoa that were genetically and morphologically distinct from *T. gallinae*. In microscopic analysis, protozoa were significantly smaller in length and width than *T. gallinae* and were bimodal in morphology. Phylogenetic analysis of the ITS1/5.8S/ITS2, *rpb1*, and hydrogenosomal Fe-hydrogenase regions revealed that the protozoan shares an ancestor with *Trichomonas vaginalis*, the sexually-transmitted agent of trichomoniasis in humans. Clinical and pathologic features of infected birds were similar to infections with *T. gallinae*. Evidence presented here strongly support taxonomical distinction of this parasite, which we hereby name *Trichomonas stableri* n. sp. This work contributes to a growing body of evidence that *T. gallinae* is not the sole etiologic agent of avian trichomonosis, and that the incorporation of molecular tools is critical in the investigation of infectious causes of mortality in birds.

## Highlights

• Trichomonad protozoa infecting free-ranging band-tailed pigeons were characterized.
• *Trichomonas gallinae* and novel species *T. stableri* were isolated in sick birds.
• *T. stableri* is significantly smaller in length and width than *T. gallinae*.
• *T. stableri* is genetically more similar to *T. vaginalis* than to *T. gallinae*.
• *T. stableri* is a secondary agent of trichomonosis in band-tailed pigeons.

## Graphical abstract

**Figure:**
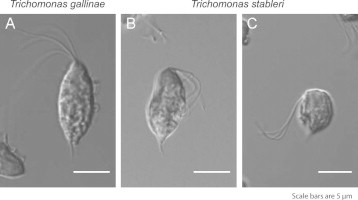

## 1 Introduction

Avian trichomonosis is an emerging and potentially fatal protozoal disease of birds characterized by the development of proliferative caseonecrotic lesions in the upper digestive tract. Worldwide, doves and pigeons (order Columbiformes) are the most common hosts of the prototypic etiologic agent, *Trichomonas gallinae*, a flagellated protozoan with an ovoid to pyriform morphology, that averages ∼7–11 μm in length (Cole, 1999; Forrester and Foster, 2008; Mehlhorn et al., 2009). Columbids are orally exposed to *T. gallinae* trophozoites through contaminated food or water, during courtship via billing, or when nestlings are fed crop milk from infected parents; raptors are exposed to *T. gallinae* via ingestion of infected prey (Stabler, 1947a; Cole, 1999). Pathology associated with *T. gallinae* infection in columbid species can range from mild to severe. In mild cases, lesions on the oral mucosa are small, superficial, and well-defined. In severe cases, lesions can be large and invasive with caseous exudate in the oropharyngeal cavity, sinuses and esophagus where they can block the passage of air and food, causing starvation or suffocation, or progress to lethal systemic infections (Stabler, 1947a; Stabler and Herman, 1951; Cole, 1999).

Trichomonosis also has been identified in a wide range of non-columbid species. Most recently, populations of passerine birds in Great Britain, Fennoscandia, France, Germany, Slovenia, and Canada have suffered *T. gallinae* epidemics (Forzán et al., 2010; Neimanis et al., 2010; Robinson et al., 2010; Gourlay et al., 2011; Lawson et al., 2012; Zadravec et al., 2012; Lehikoinen et al., 2013). In North America, mourning doves (*Zenaida macroura*) and Pacific Coast band-tailed pigeons (*Patagioenas fasciata monilis*) also experience sporadic, but seasonally associated, trichomonosis outbreaks (Stabler and Herman, 1951; Haugen and Keeler, 1952; Cole, 1999; Forrester and Spalding, 2003; Gerhold et al., 2007; Stromberg et al., 2008; USGS, 2013). Epidemiologic data and laboratory experiments indicate that trichomonad isolates from the two subspecies of band-tailed pigeons that occur in the United States (*P.f. monilis* and *P.f. faciata*) vary in virulence (Stabler and Braun, 1975).

Variation in parasite virulence is hypothesized to be related, at least in part, to the strain of the infecting organism (Stabler, 1947b, 1948; Stabler and Herman, 1951; Cole, 1999). Recent studies applying modern molecular tools have revealed that birds can be infected with diverse *T. gallinae* genotypes as well as trichomonads with only distant relatedness to *T. gallinae* including those with genetic similarity to the bovine pathogen *Tritrichomonas foetus*, or to the human pathogens *T. vaginalis* and *T. tenax* (Gerhold et al., 2008; Anderson et al., 2009; Sansano-Maestre et al., 2009; Grabensteiner et al., 2010; Lawson et al., 2011; Ecco et al., 2012; Mostegl et al., 2012; Chi et al., 2013; Lennon et al., 2013).

In the winter of 2006–2007, increased Pacific Coast band-tailed pigeon mortality in the Upper Carmel Valley of Monterey County, California was reported (Stromberg et al., 2008). Using estimates of carcasses observed over time, outbreak duration and extrapolation to suitable riparian habitat, Stromberg et al. (2008) estimated mortality during the event to be over 43,000 birds. Four live birds with suspected trichomonosis were sampled by oral swabs during the outbreak. Sequence analyses of the ITS1/5.8S rRNA/ITS2 and α-tubulin regions of the isolate genomes revealed that while one bird was infected with *T. gallinae*, the other three were infected with protozoa more similar to *T. vaginalis*, and these isolates were preliminarily categorized as belonging to the phylogenetic Sequence Group K (Gerhold et al., 2008).

A statewide surveillance program to investigate trichomonosis in band-tailed pigeons in California was initiated in 2011. Sequence analysis of the ITS1/5.8S/ITS2 region indicated that a subset of trichomonad isolates from mortality events and from live-caught birds also belonged to Sequence Group K. The present study was undertaken to characterize molecular, morphologic, epidemiologic, and pathogenic features of these avian isolates that are genetically divergent from *T. gallinae*. Herein, we provide evidence that band-tailed pigeon isolates of Sequence Group K are members of a distinct species of the Trichomonadidae family that we designate *Trichomonas stableri* n. sp.

## 2 Materials and methods

### 2.1 Bird sampling and parasite culture

Between February 2011 and August 2012, we sampled live-caught birds at backyard bird feeders, hospitalized birds at wildlife rehabilitation centers, hunter-killed birds, and dead birds involved in trichomonosis mortality events. Live birds were caught at a backyard bird feeder in Somerset, El Dorado County (38.594787, −120.596903) on May 23, 2012. Funnel entrance traps were placed on the ground under feeders where pigeons regularly fed. Captured birds were removed from the trap and placed in a plastic game bird crate or cloth bag prior to sampling and processing. Each bird was examined physically for clinical features of trichomonosis including caseous oral lesions, foul-smelling mouth odor, excess salivation, and emaciation. The oral cavity of live birds was swabbed using a cotton-tipped applicator moistened with sterile saline, and swabs were placed into InPouch™ TF culture devices (BioMed Diagnostics, White City, OR, USA). These devices can sustain cultures of *T. foetus* and other trichomonad species including *T. gallinae* (Cover et al., 1994; Bunbury et al., 2005; Gerhold et al., 2008). InPouch™ TFs inoculated in the field were kept at 25 °C during transport to the laboratory. Lindsey Wildlife Museum (Walnut Creek, California) staff was recruited to sample band-tailed pigeons admitted during the study period. Bird disposition and presence of lesions in the oral cavity were noted upon examination. The oral cavity of all band-tailed pigeons admitted to the center was sampled for trichomonad parasites prior to treatment or euthanasia. InPouch™ TFs were at ambient temperature when transported overnight to the laboratory from the rehabilitation center.

During the 2006–2007 band-tailed pigeon die-off in Monterey County, California, the oral cavities of a small subset of moribund birds with suspected trichomonosis were sampled as described previously (Gerhold et al., 2008; Stromberg et al., 2008). During winter 2011–2012, rehabilitation centers, the California Department of Fish and Wildlife (CDFW), and contacts living or working in band-tailed pigeon habitats were asked to report increased band-tailed pigeon mortality. Carcasses were collected and submitted for post-mortem examination at either the California Animal Health and Food Safety Laboratory (CAHFS, Davis, CA) or the CDFW Wildlife Investigations Laboratory (Rancho Cordova, CA). Carcasses were kept at 4 °C until shipped and samples were collected when carcasses were in fresh condition. Oral cavity, esophagus, and crop swabs obtained from dead birds were used to inoculate InPouch™ TFs. Gross pathologic findings and histologic examination of tissues were used to determine the cause of death and co-morbid conditions. Identification of caseonecrotic lesions in the upper digestive tract was suggestive of trichomonosis, and wet-mount and/or immunohistochemistry confirmed the post-mortem diagnosis of trichomonosis. Lesion tissues were sampled by excision and immediately frozen at −80 °C for molecular analysis.

InPouch™ TF cultures were incubated at 37 °C and examined once a day for 5 days by light microscopy for the presence of flagellated protozoa. Positive cultures were subcultured into Trypticase Peptone Yeast Extract Maltose (TYM) medium with 10% heat-inactivated fetal calf serum supplemented with penicillin and streptomycin (UC Davis Biological Media Services) and were subcultured for one week to generate parasites for DNA extraction and cryopreservation. Parasite culture techniques for band-tailed pigeon isolates BTPI-1 through BTPI-4 as well as mourning dove (MODO) and common ground dove (*Columbina passerine*, GRDO) isolates were described previously (Gerhold et al., 2008).

All bird capture, handling and sampling procedures were approved under the U. S. Fish and Wildlife Service Scientific Collecting Permit and the U. S. Geological Survey Federal Bird Banding Permit issued to the University of California Davis Wildlife Health Center, as well as Institutional Animal Care and Use Protocols approved by the University of California Davis.

### 2.2 Molecular characterization

DNA extraction was carried out on lesion tissues (⩽25 mg) as well as washed, pelleted axenic trophozoite cultures using the DNeasy Blood and Tissue kit (Qiagen, USA) according to the manufacturer’s protocol. DNA from three loci of the trichomonad genome: 5.8S rRNA and flanking internal transcribed spacer regions ITS1 and ITS2 (ITS1/5.8S/ITS2), the large subunit of RNA polymerase II (*rpb1*), and the housekeeping gene hydrogenosomal Fe-hydrogenase, was amplified using published primers (Felleisen, 1997; Lawson et al., 2011; Malik et al., 2011). Degenerate oligonucleotides Rpb1AF1 (5′-GAG TGT CCA GGN CAY TTY GG-3′) and Rpb1GR1 (5′-GTG GAA CGT GTT NAR NGT CAT-3′) (Malik et al., 2011) were successful in amplifying an ∼1200 bp portion of the single-copy *rpb1* gene utilized for analysis. PCR for the ITS1/5.8S/ITS2 and Fe-hydrogenase loci was carried out using HotStarTaq DNA Polymerase (Qiagen) following the manufacturer’s protocol for optimized cycling. Cycling for primers TFR1/TFR2 and TrichhydFOR/TrichhydREV utilized annealing temperatures of 65 and 53 °C, respectively. Locus *rpb1* PCR was carried out using 5 PRIME HotMasterMix (5 PRIME, Inc., USA) with an annealing temperature of 61.4 °C.

Purified PCR products (QIAquick PCR Purification Kit) were sequenced by either the DNA Sequencing Facility at UC Davis (Davis, CA), Molecular Biology Resource Facility (University of Tennessee, Knoxville) or Sequetech (Mountain View, CA). Forward and reverse sequences were assembled using Geneious Pro v. 5.3.4 (Drummond et al., 2009). Contigs were aligned to reference sequences obtained from NCBI (http://www.ncbi.nlm.nih.gov/nuccore) using MUSCLE or MAFFT (Katoh et al., 2002; Edgar, 2004) with default parameters. Pairwise genetic distance values were determined through uncorrected-*p* distance matrices generated in PAUP^∗^ (Sinaeur Associates Inc.) using prepared alignments of 260 bp for ITS1/5.8S/ITS2 and 750 bp for Fe-hydrogenase, and 1191 bp for *rpb1*. Posterior sets of phylogenetic trees (PSTs) were generated using MrBayes (Huelsenbeck and Ronquist, 2001) implemented in Geneious Pro v. 5.3.4 run for 500,000–1,000,000 generations. For the ITS1/5.8S/ITS2 alignment, the best fit nucleotide substitution model selected by MrModelTest2 v2.3 (Nylander, 2004) was a general time reversible model with gamma rate variation. For Fe-hydrogenase and *rpb1*, we applied the codon-based model of nucleotide substitution in MrBayes with gamma rate variation. Trees were sampled every 1000 generations and 25% of trees were discarded from the initial burn-in period.

### 2.3 Light microscopy

Prior to microscopic analysis, trichomonad cultures were purified by single cell cloning and axenic culture in Diamond’s medium (Diamond, 1957). Cells were passaged three times prior to morphologic analysis, including passages used for selection of the single cell clones. When cultures achieved a density of 1 × 10^6^ cells/ml, as ascertained using a hemocytometer, cells were pelleted at 2500*g* and the media removed from the pellet. Millonig’s Phosphate Buffer (0.1 M, pH 7.4) supplemented with 2.5% glutaraldehyde was then added directly to the pellet, and incubated for 2 h at 4 °C. After fixation, the cells were washed three times in Millonig’s Phosphate Buffer to remove any remaining glutaraldehyde. Cells were then allowed to settle onto poly-l-lysine coated cover slips, prior to analysis using an Olympus Fluoview 1000 Laser Scanning Confocal microscope. Due to difficulties in the out of plane focus of the flagella and cell body, depth scans were conducted, and averages of the depth scans were used for analysis. Overall, 200–220 trophozoites were measured for each isolate examined, and measurements were taken for the length of the body, width of the body, length of axostyle protrusion, and the length of the flagella. The ImageJ (Schneider et al., 2012) software package was used to accurately measure the dimensions of the trophozoites. After analysis, the significance of the data was evaluated using a Student’s *t*-test with a *P*-value of <0.05 indicating a significant difference.

### 2.4 Immunohistochemistry

Formalin-fixed paraffin-embedded tissues were sectioned at 4 μm, placed on charged slides and allowed to dry overnight. The slides were deparaffinized with xylene and rehydrated through graded alcohols with a 3% H_2_O_2_ in methanol peroxidase blocking step following the absolute alcohol. The slides were incubated in 0.1% protease from *Streptomyces griseus* (Sigma, P5147) at 37 °C for 15 min. After rinsing in water and then Tris-buffered saline/Tween 20 (TBS-T), the slides were blocked for 10 min at room temperature with 0.5% casein in TBS-T. A rabbit polyclonal directed against *T. foetus* (produced at CAHFS-UCD) was applied to the slides for 30 min at room temperature. After rinsing with TBS-T, HRP-labeled rabbit polymer (Dako, Envision+, K4002) was incubated on the slides at room temperature for 30 min. Following a TBS-T rinse, the chromogen AEC (Dako, K3464) was applied for 10 min at room temperature. After rinsing, the slides were counterstained using Mayer’s Hematoxylin and blued. Aqueous mounting medium is applied and allowed to harden and then coverslips were applied with permanent mounting medium.

## 3 Results

As part of an epidemiological investigation into free-ranging Pacific Coast band-tailed pigeon population health, and ongoing efforts to monitor large-scale band-tailed pigeon mortality in the central coast region of California, we isolated parasite DNA and cultivated *Trichomonas* sp. protozoa from infected birds (Table 1). In an effort to clarify the relationship between the prototypical agent of avian trichomonosis, *T. gallinae*, and a species that clustered more closely with *T. vaginalis* in preliminary sequence analysis of the ITS1/5.8S/ITS2 region, we performed morphological, molecular and post-mortem comparisons. Results from these experiments indicate the presence of two trichomonad species infecting band-tailed pigeons during trichomonosis epidemics and during the breeding season: *T. gallinae*, and a similarly pathogenic, but genetically and morphologically distinct species which we are naming *T. stableri*.

The majority of *T. stableri*-infected birds were adults that died during the 2006–2007 or 2012 mortality events in the winter and early spring in Monterey County, California (*n* = 7) and Madera County, California (*n* = 1) (Table 1). *T. stableri* also was isolated from a clinically healthy bird that was live-caught in El Dorado County, California on May 23, 2012, and another *T. stableri*-infected bird with clinical signs consistent with trichomonosis was submitted for rehabilitation on February 18, 2012 at the Lindsay Wildlife Museum in Walnut Creek, Contra Costa County, California, where it died three days later (Table 1). Because of the overlapping time period of this latter submission with ongoing mortality events in nearby Monterey County, this bird may have been infected during epidemic transmission prior to migration to Contra Costa County.

### 3.1 Morphological analysis

**Fig. 1:**
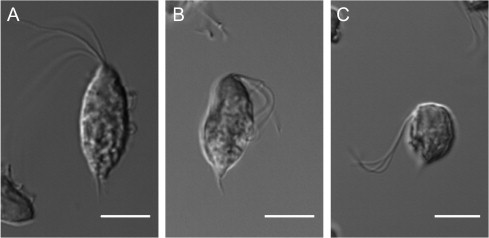
Micrographs of *T. gallinae* and *T. stableri* trophozoites. (A) *T. gallinae,* illustrating the four anterior flagella, and the extension of the axostyle. Note the slender appearance of the trophozoite with a near elliptical form. (B) Slender form of *T. stableri* is shown with protruding axostyle and four anterior flagella. (C) Rounded form of *T. stableri* is shown with the four anterior flagella. All scale bars are 5 μm.

Using light microscopy, we compared the morphologies of *T. gallinae* (isolate BTPI-1) and *T. stableri* (BTPI-3) isolated during a trichomonosis epidemic in Pacific Coast band-tailed pigeons of Carmel Valley, California (Gerhold et al., 2008; Stromberg et al., 2008). Based on analysis of 200 trophozoites, it was determined that *T. gallinae* BTPI-1 had a length of 14.18 ± 1.48 μm and a width of 7.32 ± 0.81 μm (Fig. 1A). It should be noted that while other studies have observed polymorphisms in the trophozoite shape of *T. gallinae* (Mehlhorn et al., 2009) with wide ranges in parasite length and width (e.g., length range of 6.2–18.9 μm, and a width range of 2.3–8.5 μm) (Stabler, 1941), *T. gallinae* isolate BTPI-1 displayed no such diversity in morphology. In contrast, analysis of 220 *T. stableri* trophozoites revealed that protozoa of this species had a bimodal distribution in parasite measurements. A slender form of *T. stableri* isolate BTPI-3 measured 12.98 ± 0.85 μm in length and 5.95 ± 0.94 μm in width (Fig. 1B) and a rounded form of *T. stableri* measured 8.09 ± 0.79 μm in length and 5.88 ± 0.64 μm in width (Fig. 1C). There was no change in the proportion of slender vs. wide forms during early or late stages of culture. Polymorphisms among trophozoites may be explained by a lack of cell cycle synchrony. As seen by scanning electron microscopy, trichomonad cells in the process of dividing have varying morphologies as they move through each cycle step (Benchimol, 2004).

*T. gallinae* BTPI-1 was significantly longer in length compared to slender forms (*P *= 0.004) and rounded forms (*P < *0.001) of *T. stableri*, and also was significantly wider compared to slender forms (*P *< 0.001) and rounded forms (*P *< 0.001) of *T. stableri*. There was a significant difference in the length of the slender and round forms of *T. stableri* (*P *= 9.37 × 10^−30^), but no difference in width (*P *= 0.49). Further analysis of the trophozoite morphology indicated that there were no differences in the length of the flagella between *T. gallinae* BTPI-1 and *T. stableri* BTPI-3, which had a mean measurement of 13.87 ± 3.23 μm, and axostyles of each species had a similar length of protrusion.

### 3.2 Molecular analysis

#### 3.2.1 ITS1/5.8S/ITS2

All 10 *T. stableri* parasite isolates listed in Table 1 were nearly identical across the ITS1/5.8S/ITS2 region. Sequences from isolates BTPI-2, -3, and -4 collected during the 2006–2007 Monterey County mortality event (GenBank EU215367, 322 bp, Sequence Group K) were identical to isolates collected from band-tailed pigeons in 2012 including CA005890, CA015506, CA015499, CA015497 and CA015840 (GenBank KC215389, 367 bp) across overlapping regions (Table 1). Two isolates, CA015496 and CA015500 (GenBank KC215390, 367 bp), were different by a single nucleotide (C → T) at position 341 based on the alignment of GenBank sequences KC215389 and KC215390.

**Fig. 2:**
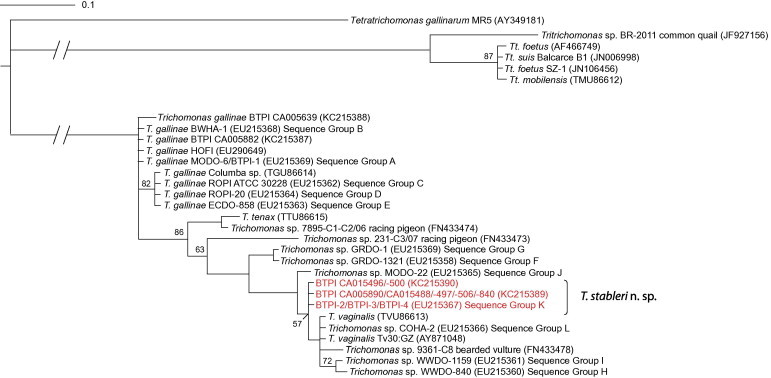
Consensus phylogenetic tree of ITS1/5.8S/ITS2 sequences (260 bp alignment) made by Bayesian inference. Legend denotes substitutions per site. All posterior probabilities are >90% unless otherwise noted. ITS region Sequence Groups refer to designations described in Gerhold et al. (2008). BTPI, band-tailed pigeon; BWHA, broad-winged hawk; COHA, Cooper’s hawk; ECDO, Eurasian collared dove, GRDO, common ground dove; HOFI, house finch; MODO, mourning dove; ROPI, rock pigeon; WWDO, white-winged dove.

We aligned ITS1/5.8S/ITS2 sequences of select band-tailed pigeon parasite isolates listed in Table 1 with representative sequences of the Trichomonadidae family available in GenBank for pairwise distance calculation and phylogenetic analysis. Trimming the alignment to 260 bp removed the single polymorphic nucleotide position that distinguished sequence KC215390 from KC215389. Bayesian phylogenetic inference revealed that *T. stableri* isolates shared a common ancestor with human *T. vaginalis* isolates and avian *Trichomonas* spp. isolates from common ground doves, a mourning dove, a white-winged dove (*Zenaida asiatica*, WWDO), and a Cooper’s hawk (*Accipiter cooperii*, COHA), which were all from the U.S., as well as a bearded vulture (*Gypaetus barbatus*) from the Czech Republic (Fig. 2). Although the clade containing band-tailed pigeon sequences KC215390, KC215389, EU215267, *T. vaginalis* and avian *T. vaginalis*-like sequences formed a polytomy with only 57% posterior probability, *T. stableri* isolates were clearly distinguishable from *T. gallinae*, *T. vaginalis* and other avian *T. vaginalis*-like species (Fig. 2). Lack of resolution at some nodes of the phylogenetic tree is most likely related to the short sequence length and highly conserved regions of the locus.

Using the same sequence alignment to derive genetic distance between isolates at the ITS1/5.8S/ITS2 locus, *T. stableri* isolates had 99.6% pairwise similarity compared to *T. vaginalis* isolates from humans and a *Trichomonas* sp. isolate from a Cooper’s hawk (COHA-2, Sequence Group L), and 99.2% and 98.3% pairwise similarity to *Trichomonas* sp. isolates from a mourning dove (MODO-22, Sequence Group J) and a bearded vulture, respectively. *T. stableri* isolates were more distantly related to common ground dove isolates GRDO-1 and GRDO-1321 belonging to Sequence Groups G and F, respectively (96.3% pairwise similarity). Pairwise similarity of *T. stableri* sequences compared to *T. gallinae* sequences ranged from 92.1% to 93.4% in the ITS1/5.8S/ITS2 alignment. Notably, *T. gallinae* isolates collected from dead or dying band-tailed pigeons (BTPI-1 and BTPI CA005882) during the Carmel Valley, California die-offs in 2006–2007 and 2012 had only 92.6% pairwise similarity compared to *T. stableri* isolates collected from birds at the same events.

#### 3.2.2 Fe-hydrogenase

**Fig. 3:**
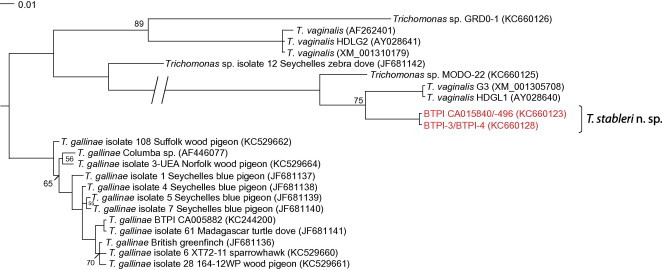
Consensus phylogenetic tree of partial Fe-hydrogenase gene sequences (750 bp alignment) made by Bayesian inference. Legend denotes substitutions per site. All node posterior probabilities are >90% unless otherwise noted.

We aligned *Trichomonas* spp. Fe-hydrogenase sequences available in GenBank with those generated in this study from *T. gallinae* (KC244200), *T. stableri* (KC660123/KC660124/KC660128), MODO-22 (KC660125) and GRDO-1 (KC660126) for pairwise genetic distance calculation and phylogenetic analysis. In the 750 bp alignment, band-tailed pigeon sequences from birds CA015496, CA015840, BTPI-3 and BTPI-4 which were infected with *T. stableri* formed a sister clade to *T. vaginalis* (HDGL1 gene), supported by a posterior probability of 75% (Fig. 3). Isolate MODO-22 was most closely related to *T. vaginalis* and *T. stableri* isolates, while GRDO-1 clustered with HDGL2 gene sequences of *T. vaginalis*. *T. gallinae* Fe-hydrogenase sequences of protozoa isolated from birds in Africa, North America and Europe formed a strongly-supported monophyletic clade that included isolates implicated in avian trichomonosis outbreaks in both Great Britain (e.g., JF681136) and California (e.g., KC244200). In an uncorrected-*p* distance matrix of the same 750 bp alignment, *T. stableri* sequences had 94.1% pairwise similarity to human *T. vaginalis* sequences (XM_001305708 and AY028640), 93.5% similarity to MODO-22, and only 82.1% similarity to *T. gallinae* isolated from a band-tailed pigeon sampled during a trichomonosis outbreak (BTPI CA005882, GenBank KC244200).

Amplification of the Fe-hydrogenase gene by PCR was not always successful in bird tissue samples, perhaps due to DNA degradation during decomposition or to PCR inhibition by tissue components. Inconsistent PCR results led us to believe that two birds sampled at trichomonosis outbreaks (CA015497 and CA005890) were co-infected with *T. stableri* and *T. gallinae*. In both cases, ITS1/5.8S/ITS2 and *rpb1* PCR amplified DNA identical to *T. stableri*, whereas, in the Fe-hydrogenase locus, sequences were identical to *T. gallinae* (Table 1). In isolates from two birds (CA015840, CA015496), Fe-hydrogenase sequencing revealed the presence of a nucleotide mixture (A/T) at position 634 (based on 915 bp alignment of GenBank KC660123 and KC660124), conferring amino acids isoleucine and leucine. At the same position, *T. stableri* isolates BTPI-3 and BTPI-4 (KC660128) from the 2006 to 2007 Carmel Valley mortality event encoded an isoleucine, while *T. vaginalis* and all other *Trichomonas* sp. sequences analyzed in the study encoded a leucine.

#### 3.2.3 *Rpb1*

To further examine the genetic relatedness of *T. stableri* to avian and human trichomonad parasites, we amplified an ∼1200 bp region of the single copy gene encoding the largest subunit of eukaryotic RNA polymerase II, *rpb1*, from trichomonad isolates collected from band-tailed pigeons during the 2012 trichomonosis outbreak in Carmel Valley, California. *T. stableri* isolates CA005890, CA015497 and CA015840 were identical across overlapping regions, and the sequence with the longest read, CA015840, was deposited in GenBank (KF233590, 1200 bp). Similarly, *T. gallinae* isolates CA005950 and CA015554 were identical across the amplified *rpb1* region, and the sequence from isolate CA005950 was deposited in GenBank (KF233589, 1185 bp).

**Fig. 4:**
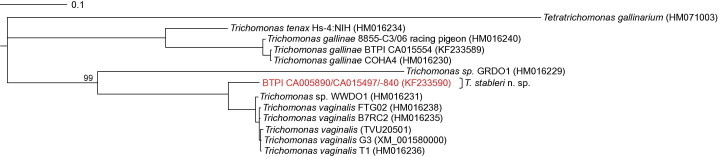
Consensus phylogenetic tree of partial *rpb1*gene sequences (1191 bp alignment) made by Bayesian inference. Legend denotes substitutions per site. All node posterior probabilities are 100% unless otherwise noted.

Representative nucleotide sequences from each species were aligned to relevant *rpb1* sequences available in GenBank resulting in a 1188 bp alignment that was trimmed to the proper open reading frame. In the consensus Bayesian phylogenetic tree, *T. stableri* clustered with a *Trichomonas* sp. white-winged dove isolate (WWDO 1200, Gerhold et al. 2008) and *T. vaginalis* isolates (Fig. 4) in a monophyletic clade whose node was supported by a posterior probability of 100%. The closest ancestor to this clade was a *Trichomonas* sp. protozoan isolated from a common ground dove (GRDO-1) (Fig. 4). *T. gallinae rpb1* gene sequences were most closely related to *T. tenax*, and with the *T. tenax* sequence formed a monophyletic clade that was genetically distant from the common ground dove/*T. stableri*/*T. vaginalis* clade (Fig. 4). At the *rpb1* locus, *T. stableri* had a pairwise similarity of 93.2% to isolate WWDO 1200 and 93% similarity to *T. vaginalis*, but only 75.1–75.3% pairwise similarity to *T. gallinae* and 75.5% similarity to *T. tenax*.

### 3.3 Pathology

**Fig. 5:**
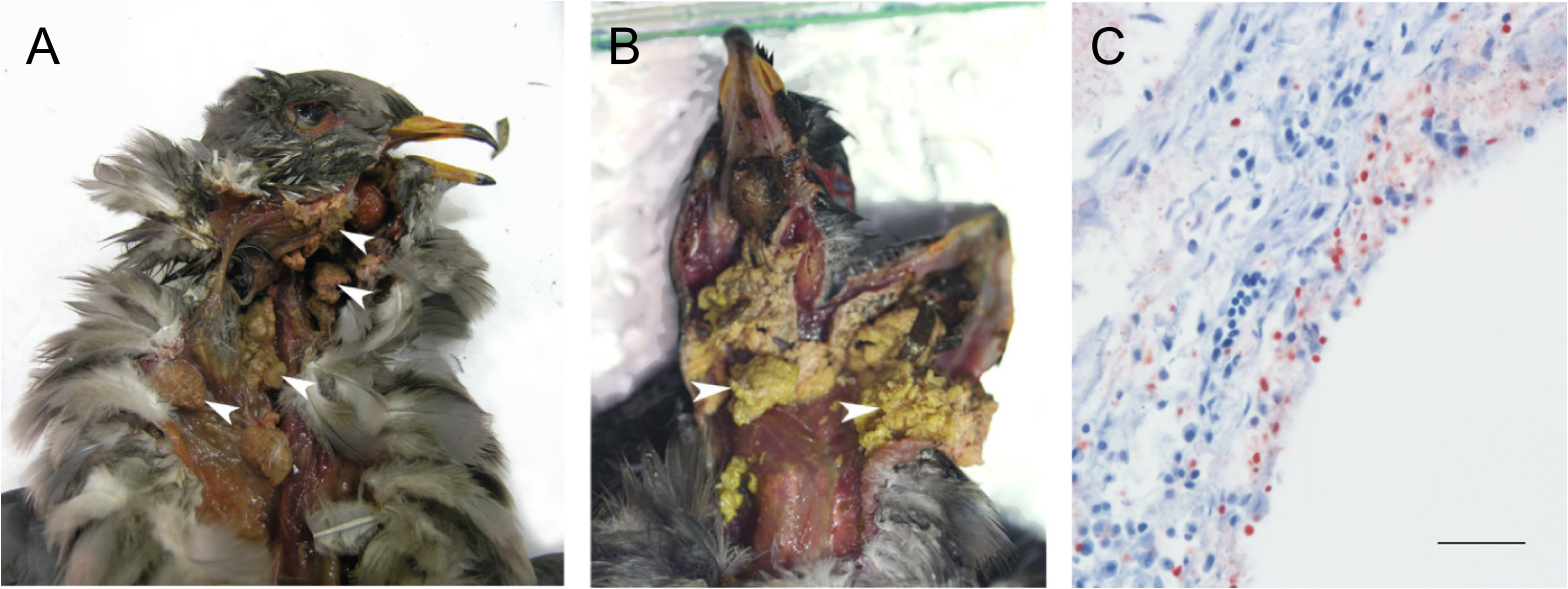
Pathogenesis of *T. stableri* in Pacific Coast band-tailed pigeons. Oral and esophageal caseonecrotic lesions (e.g., white arrowheads) observed during post-mortem examination in cases CA015500 (A) and CA015499 (B). Both birds were collected and sampled between February 16 and February 19, 2012 during a trichomonosis mortality event in Monterey County, California. Infection with *T. stableri* was confirmed by DNA amplification performed directly on lesion tissues. (C) Immunohistochemical staining of trichomonad antigen (red) in lung tissue of case CA015506. Lung infection likely occurred via aspiration of necrotic debris from the laryngeal region. Scale bar is 50 μm. (For interpretation of the references to colour in this figure legend, the reader is referred to the web version of this article.)

Post-mortem examination of Pacific Coast band-tailed pigeons that died during trichomonosis mortality events or at the rehabilitation center facilitated a comparison of the pathogenesis of *T. gallinae* and *T. stableri*. Putative co-infections in cases CA005890 and CA015497 precluded our ability to assess the role of individual protozoan strains in disease pathogenesis in these birds. Based on sequence analysis of the ITS1/5.8S/ITS2 and Fe-hydrogenase regions in parasite DNA extracted directly from lesion tissues, band-tailed pigeons CA015506, CA015499, CA015496, and CA015500 were most likely singly-infected with *T. stableri*. All four birds had severe caseonecrotic oropharyngitis and cellulitis, with lesions present in the oral cavity that extended into the esophagus (Fig. 5A and B). Histological examination revealed that lesions contained both protozoa and bacteria. Bird CA015506 had caseonecrotic lesions obstructing the larynx as well as severe, focally extensive necrotizing pneumonia with intralesional bacteria which likely occurred following aspiration of necrotic material containing bacteria. Immunohistochemical staining of lung tissues in bird CA015506 confirmed infection with trichomonad protozoa (Fig. 5C).

### 3.4 Taxonomic summary

*Host:* Pacific Coast band-tailed pigeon (*Patagioenas fasciata monilis*).

*Primary site of infection:* Oral cavity, esophagus.

**Fig. 6:**
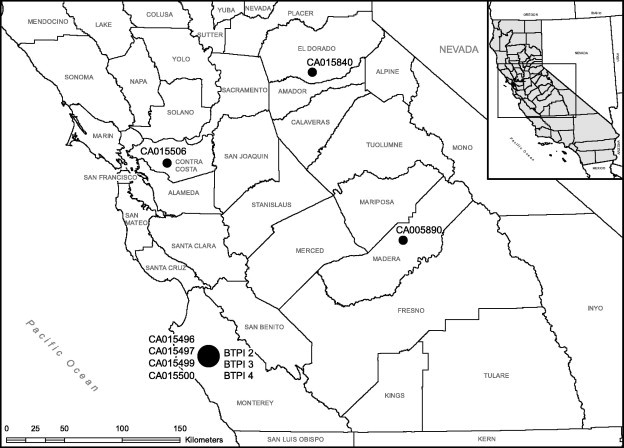
Geographic distribution of birds (solid black circles) infected with *T. stableri* in California. Circle size is relative to the number of birds infected at each site.

*Locality:* Central, Central Coast, and Sierra Nevada regions of California (See Fig. 6 for isolate map).

*Morphology:* Slender form: *n* = 107, range (length = 11.02–15.06 μm, width = 5.29–7.93 μm); round form: *n* = 113, range (length = 7.58–9.12 μm, width = 5.09–7.32 μm).

*GenBank accession numbers:* KC215389, KC215390, EU215367 (ITS/5.8S/ITS2); KC660123, KC660124, KC660128 (Fe-hydrogenase); KF233590 (*rpb1*).

*Etymology:* The Latin name of Stabler is used for a species name. Dr. Robert M. Stabler was a pioneer in the field of avian trichomonosis research in columbids of North America.

## 4 Discussion

Herein, we describe morphologic, genetic, and pathogenic characteristics of a secondary agent of avian trichomonosis in Pacific Coast band-tailed pigeons, and propose that the organism be named *T. stableri*. Sequence analyses across multiple loci demonstrate that *T. stableri* is most closely related to *T. vaginalis*, the etiologic agent of human trichomoniasis, and the phylogenetic positions of these two species indicate that they evolved from a common ancestor. The relatedness of *T. stableri* and other avian trichomonads to *T. vaginalis* (Gerhold et al., 2008; Grabensteiner et al., 2010) is intriguing and suggests that one or more host species jump events occurred in the past. The growing list of genetically similar trichomonads isolated from ecologically distinct hosts highlights the continued need to study the genetic relatedness and transmission potential of trichomonad protozoa across species (Okamoto et al., 1998; Kutisova et al., 2005; Duboucher et al., 2006; Zalonis et al., 2011; Reinmann et al., 2012; Walden et al., 2013).

Thus far, *T. stableri* has only been identified in the Pacific Coast band-tailed pigeon, which is a migratory upland game species distributed in coastal and inland mixed hardwood and coniferous forests between southeastern Alaska and northern Baja California (Sanders, 2012). The North American Breeding Bird Survey, Mineral Site Survey, and other sources indicate that this population has been in decline for the past 40 years (Keppie and Braun, 2000; Sanders, 2012). During ongoing investigations into significant causes of morbidity and mortality in this species in California, we detected both *T. stableri* and *T. gallinae* (sometimes as a co-infection) during epidemic periods of trichomonosis, and we hypothesize that recurrent epidemics involving both agents are contributing to the population’s decline. Further sampling and genetic analysis will be critical to improving our understanding of the evolutionary history of *T. stableri*, particularly as it relates to *T. gallinae* and other members of the Trichomonadidae family found in wildlife and humans.

The *in vivo* phenotype and host range of *T. stableri* and other avian *T. vaginalis*-like strains in columbids and spill-over species require additional characterization, particularly given evidence presented here that *T. stableri* is associated with large-scale avian mortality. Band-tailed pigeons infected with *T. stableri* in Monterey County during 2012 outbreaks had severe caseous obstructive lesions indistinguishable from those caused by *T. gallinae*. Trichomonads belonging to ITS1/5.8S/ITS2 Sequence Group L, which has 99.6% similarity to *T. stableri* (formerly Sequence Group K), were also isolated during trichomonosis epidemics in Cooper’s hawks and mourning doves in Arizona (USA) (Gerhold et al., 2008). At the same time, the pathogenic potential of parasites belonging to Sequence Group L in white-winged doves in Texas (USA) and the *T. vaginalis*-like species in a bearded vulture in Central Europe remain unclear (Gerhold et al., 2008; Grabensteiner et al., 2010). Field and laboratory studies that further explore the relationship between parasite genotype, disease pathogenesis and avian trichomonosis epidemiology will improve our understanding of the impact of *T. stableri* and other *T. vaginalis*-like species on susceptible avian populations around the world.

## Figures and Tables

**Table 1:** *Trichomonas stableri* and *T. gallinae* culture and DNA isolates collected from Pacific Coast band-tailed pigeons.

Case ID	Infecting *Trichomonad* species	Collection location	Age/sex	Sample type	Collection date	Bird cycle season	Surveillance type	Oral lesions	GenBank accession No. (sample type)		
									ITS1/5.8S/ITS2	Fe-hydrogenase	rpb1
BTPI-1	*T. gallinae*	Carmel Valley, Monterey Co.	UNK	O	2/14/2007	Winter/ESM	Mortality Event	ND	EU215369	KC660127	ND
BTPI-2	*T. stableri*	Carmel Valley, Monterey Co.	UNK	O	2/14/2007	Winter/ESM	Mortality Event	ND	EU215367	PCR Fail	ND
BTPI-3	*T. stableri*	Carmel Valley, Monterey Co.	UNK	O	2/14/2007	Winter/ESM	Mortality Event	ND	EU215367	KC660128	ND
BTPI-4	*T. stableri*	Carmel Valley, Monterey Co.	UNK	O	2/14/2007	Winter/ESM	Mortality Event	ND	EU215367	KC660128	ND
CA015554	*T. gallinae*	Coarsegold, Madera Co.	AF	O & T	1/17/2012	Winter/ESM	Mortality Event	Yes	KC215387 (O & T)	KC244200 (O)	KF233589 (O)
CA005882	*T. gallinae*	Carmel Valley, Monterey Co.	AM	O	1/19/2012	Winter/ESM	Mortality Event	Yes	KC215387	KC244200	ND
CA005890	*T. stableri*/*T. gallinae*	Coarsegold, Madera Co.	AM	O	1/19/2012	Winter/ESM	Mortality Event	Yes	KC215389	KC244200 (*T. gallinae*)	KF233590
CA015500	*T. stableri*	Carmel Valley, Monterey Co.	AM	T	2/16/2012	Winter/ESM	Mortality Event	Yes	KC215390	PCR Fail	ND
CA015506	*T. stableri*	Walnut Creek, Contra Costa Co.	AM	T	2/18/2012	Winter/ESM	Found Sick^a^	Yes	KC215389	PCR Fail	ND
CA015499	*T. stableri*	Carmel Valley, Monterey Co.	AF	T	2/19/2012	Winter/ESM	Mortality Event	Yes	KC215389	PCR Fail	ND
CA015496	*T. stableri*	Carmel Valley, Monterey Co.	AF	O & T	2/21/2012	Winter/ESM	Mortality Event	Yes	KC215390 (O & T)	KC660123 & KC660124 (O & T)	ND
CA015497	*T. stableri/T. gallinae*	Carmel Valley, Monterey Co.	AF	O & T	2/21/2012	Winter/ESM	Mortality Event	Yes	KC215389 (O)	KC244200 (*T. gallinae*) (T)	KF233590 (O)
CA015840	*T. stableri*	Somerset, El Dorado Co.	AF	O	5/23/2012	Breeding & Nesting	Live-caught	No	KC215389	KC660123 & KC660124	KF233590

O, culture isolate from oral swab; T, DNA isolate from lesion tissue; AM, adult male; AF, adult female; NA, not applicable; ND, no data; UNK, data unknown; ESM, early spring migration.

a Sampled at the Lindsay Wildlife Museum, Walnut Creek, California.
